# A comparative, observational study evaluating dosing characteristics and ovarian response using the recombinant human follicle-stimulating hormone pen injector with small-dose dial in assisted reproductive technologies treatment in Asia: IMPROVE study

**DOI:** 10.1186/s12958-021-00882-2

**Published:** 2022-01-17

**Authors:** Bum Chae Choi, Canquan Zhou, Hong Ye, Yun Sun, Ying Zhong, Fei Gong, Ivan Sini, Nadezda Abramova, Salvatore Longobardi, Miranda Hickey, Thomas D’Hooghe

**Affiliations:** 1 Creation and Love Women’s Hospital, Gwang-ju, Korea; 2 First Affiliated Hospital, SunYat-sen University, GuangZhou, Guangdong China; 3 Chongqing Maternity and Child Healthcare Hospital, Chongqing, China; 4 Center for Reproductive Medicine, Ren Ji Hospital, School of Medicine, Shanghai Jiao Tong University, Shanghai, China; 5 Chengdu Jinjiang District Maternal and Child Health Hospital, Chengdu Shi, China; 6 Reproductive and Genetic Hospital of CITIC-Xiangya, Changsha, Hunan China; 7 Indonesian Reproductive Science Institute (IRSI), Morula IVF, Jakarta, Indonesia; 8 Merck Healthcare KGaA, Darmstadt, Germany; 9 Merck Healthcare Pty. Ltd (an affiliate of Merck KGaA), NSW Macquarie Park, Australia; 10 Research Group Reproductive Medicine, Department of Development and Regeneration, Organ Systems, Group Biomedical Sciences, KU Leuven (University of Leuven), Leuven, Belgium; 11 Department of Obstetrics and Gynecology, Yale University, New Haven, CT USA

**Keywords:** Recombinant human follicle-stimulating hormone/r-hFSH pen injector, ART, OHSS

## Abstract

**Background:**

Ovarian stimulation during medically assisted reproduction treatment should be individualized to optimize outcomes and reduce complications. This study assessed whether use of the recombinant human follicle-stimulating hormone (r-hFSH) pen injector allowing small 12.5 IU dose increments resulted in lower r-hFSH dose per oocyte retrieved in a subgroup of patients at risk of OHSS, compared with r-hFSH injection devices allowing only 37.5 IU increments.

**Methods:**

This multicenter, comparative, observational study evaluated patients from a prospective (study group) and historical (control group) cohort. The study group enrolled 1783 patients using the redesigned r-hFSH pen injector (GONAL-f®, Merck Healthcare KGaA, Darmstadt, Germany) from a prospective phase IV, non-interventional, open-label study, conducted in Korea, Vietnam, Indonesia, and China. The control group consisted of 1419 patients from a historical study using r-hFSH devices allowing 37.5 IU increments. In the study group, 397 patients were considered at risk of OHSS; this information was unavailable for the control group, so biomarkers and patient characteristics were used to match 123 patients from the study group and control group. Each center adhered to standard practice; starting dose and intra-cycle dose adjustments were allowed at any point. The primary endpoint, amount of r-hFSH (IU) administered per oocyte retrieved, was assessed in matched patients only. Additional outcomes and safety were assessed in the overall populations.

**Results:**

Baseline characteristics were comparable between groups. Mean (SD) total dose of r-hFSH administered per oocyte retrieved in patients at risk of OHSS, was significantly lower in the study group compared with the control group (132.5 [85.2] vs. 332.7 [371.6] IU, *P* < 0.0001, n = 123). Implantation rate, clinical pregnancy rate, and live birth rates in the overall study and control groups were 30.0 vs. 20.6%, 50.3 vs. 40.7%, and 43.8 vs. 34.0%, respectively. OHSS incidence was significantly lower in the study group compared with the control group (27/1783 [1.5%] vs. 57/1419 [4.0%] patients, *P* < 0.0001). AEs were reported by 5.0% of patients in the study group.

**Conclusions:**

A significantly lower r-hFSH dose per oocyte retrieved and lower OHSS incidence were observed in patients using the redesigned injector compared with patients using other injection devices.

**Supplementary Information:**

The online version contains supplementary material available at 10.1186/s12958-021-00882-2.

## Introduction

Recombinant human follicle-stimulating hormone (r-hFSH-alfa [GONAL-f®, Merck Healthcare KGaA, Darmstadt, Germany]) is used for controlled ovarian stimulation (COS) during assisted reproductive technologies (ART) treatment [1]. As stipulated in the prescribing information, ‘*a commonly used regimen for superovulation involves the administration of 150–225 IU of GONAL-f daily, commencing on days 2 or 3 of the cycle. Treatment is continued until adequate follicular development has been achieved (as assessed by monitoring of serum oestrogen concentrations and/or ultrasound examination), with the dose adjusted according to the patient’s response, to usually not higher than 450 IU daily*’ [2]. Therefore, there is considerable scope for the personalization of treatment, with the opportunity to individualize the starting dose and to adjust the dose during ovarian stimulation.

Starting dose is commonly individualized in order to optimize treatment efficacy and safety [3, 4]. Patient characteristics and ovarian reserve biomarkers, including age, body mass index (BMI), antral follicle count (AFC), anti-Müllerian hormone (AMH) concentration, Day 3 FSH, and response to any previous ovarian stimulation cycle have been used to predict ovarian response as part of the pre-stimulation management and are usually used to assist the selection of appropriate protocol and gonadotropin starting dose [3, 5–7]. Some patients, especially those with a predicted hyper-response to gonadotropin treatment, have an increased risk of ovarian hyperstimulation syndrome (OHSS); therefore, individualization of the r-hFSH starting dose could help to minimize this risk [8–11].

During treatment, a patient’s response to ovarian stimulation and follicular development are closely monitored by ultrasound assessment and hormonal testing [7, 12]. In patients with unexpected hyper-ovarian response, reduction of gonadotropin dose during the stimulation cycle (i.e., intra-cycle dose adjustment) may reduce the likelihood of moderate and severe OHSS [13]. In patients with unexpected low ovarian response, an increase in gonadotropin dose during the stimulation cycle can reduce the risk of cycle cancellation due to inadequate response [14, 15]. Intra-cycle dose adjustments are common in clinical practice [16]; however, the dosing characteristics in specific patient sub-populations, for example those with increased risk of OHSS, are not well described in clinical research papers.

Choosing an appropriate r-hFSH starting dose and adapting that dose during treatment to meet individual patients’ needs are both important aspects of individualized ART treatment. The GONAL-f® (r-hFSH; follitropin alfa; Merck Healthcare KGaA, Darmstadt, Germany) pen injector, which has recently been introduced in several Asian countries, allows small dose increments of 12.5 IU. This feature may be particularly advantageous in terms of the selection of gonadotropin starting dose and intra-cycle dose adjustments as it allows a dose to be fine-tuned to a greater extent than older GONAL-f devices with ≥37.5 IU dose dial increments.

The usability of this prefilled GONAL-f pen, allowing small dose 12.5 IU increments, has been confirmed in human factors engineering studies assessing dose accuracy, handling, and readability [17, 18]. Furthermore, the ease of use and handling errors were assessed in questionnaire-based and simulated use studies, with the GONAL-f pen injector preferred over other injection devices by women with recent or current infertility, and by fertility nurses [19–22]. In addition, there is a growing body of evidence confirming the value of gonadotropin injector devices allowing subtle dose changes with respect to reducing the risk of OHSS [9–11]. Nevertheless, there is a need for studies assessing whether the use of such injectors would lead to improved treatment efficacy and clinical outcomes, specifically in patients at increased risk of OHSS.

The aim of the IMPROVE study was to evaluate whether the introduction of the pen injector, which allows fine-tuning of the gonadotropin dose, leads to a reduction in total r-hFSH dose per oocyte retrieved in Asian patients at risk of OHSS, when compared to the use of other r-hFSH injection devices.

## Materials and methods

### Study design

This was a multicenter, comparative, observational study evaluating patients from a prospective cohort (study group) and a historical cohort (control group). Patients in the study group used the r-hFSH pen injector, which allowed small dose adjustments in 12.5 IU increments. Patients in the control group used other r-hFSH injection devices, including an older model of the r-hFSH pen injector which allowed only 37.5 IU increments. Patients from the study group participated in a prospective study (conducted between 11 September 2014 and 29 July 2016) with a 24-month recruitment period, a ~2-month treatment period, and a 10-month observation period. Patients from the control group had participated in an older phase IV, prospective, multicenter, observational study (conducted between 14 June 2010 and 29 February 2012).

### Study population and recruitment

The study group included patients at 14 sites across China, Indonesia, Korea, and Vietnam. The control group included patients from 34 centers in nine countries, including China, India, Indonesia, Korea, Malaysia, Pakistan, Singapore, Thailand, and Vietnam. Visits, treatment, and diagnostic procedures, including collection of data for biomarkers and baseline characteristics, were performed according to the local routine clinical practice, and there were no additional interventions or laboratory tests other than routine practice performed specifically for this study. Inclusion and exclusion criteria for the study and control groups are described in Table 1.

**Table 1:** Inclusion/exclusion criteria of the study and control groups

	Study group	Control group
Age (years)	20–40	20–40
Both ovaries present	Yes	Yes
Gonadotropin administration method	Redesigned GONAL-f pen injector allowing 12.5 IU dose increments	Other GONAL-f delivery methods^a^
Without polycystic ovarian syndrome	Yes	Yes
BMI (kg/m^2^)	<30	<30
No use of urinary hMG/FSH or clomiphene citrate in same cycle	Yes	Yes
No contraindication to GONAL-f according to local SmPC	Yes	Yes

*hMG* human menopausal gonadotropin; *FSH* follicle-stimulating hormone; *SmPC* Summary of Product Characteristics

^a^Including older model of r-hFSH pen injector which allowed 37.5 IU dose increments

For the primary analysis, patients at risk of OHSS were identified in the study group by the investigators at each study site using potential risk factors that included patient characteristics (BMI and age) and ovarian reserve biomarkers at baseline (AMH, AFC, FSH, luteinizing hormone [LH], and estradiol [E2]). The final decision on whether a patient was at risk of OHSS was made by the investigator. The classification of patients at risk of OHSS was not available for the control group, however, available data for the above mentioned patient characteristics and biomarkers allowed matching patients at risk of OHSS from the study group to patients from the control group on the basis of finding the nearest match.

### Treatment

Patients included in the study and control groups were from non-interventional observational studies; therefore, individualization of the starting dose and dose adjustments during ovarian stimulation were done according to routine practice and at the discretion of the treating physicians at each of the participating centers. The individualization of the starting dose was performed at the start of the treatment. r-hFSH dose adjustments during ovarian stimulation were allowed at any stage in both cohorts. In the study group, patients received either a long GnRH agonist or a GnRH antagonist protocol, whereas all patients in the historical cohort received a long GnRH agonist protocol, which was consistent with clinical practice during the two study periods.

### Endpoints

The primary endpoint was the mean total dose of r-hFSH (IU) administered per oocyte retrieved and was evaluated in matched pairs of patients at risk of OHSS in the study and control groups. Since the study group included patients using a GnRH agonist or the antagonist protocols and the patients in the control group were using only a GnRH agonist protocol, the sensitivity analysis was conducted for the primary outcome excluding patients using a GnRH antagonist protocol. The primary analysis and sensitivity analysis were not adjusted for patients who used r-hFSH as part of the 2:1 r-hFSH:r-hLH combination therapy using a single pen injector (Pergoveris^®^; follitropin alfa/lutropin alfa; Merck Healthcare KGaA, Darmstadt, Germany). A separate analysis of significant factors to predict risk for OHSS was performed using patients from the overall study and control groups combined.

Secondary endpoints were exploratory and were evaluated for all eligible patients from the study and control groups, without statistical analysis to compare these outcomes between the two groups. Secondary outcomes included: initial, total, and daily mean doses of r-hFSH and treatment duration; serum E2 level on the day of hCG administration; clinical pregnancy, implantation, cycle cancellation, live birth rates, and number of multiple pregnancies. For the study group, details of dose adjustments of <37.5 IU, including frequency, sequence, and stimulation day were also assessed.

Usability and quality of life analysis with the redesigned pen injector were assessed and reported for the study group. An optional patient questionnaire was provided to the patients during clinic visits. The patient questionnaire was designed by the Medical Lead at the time that the prospective study was conducted (TU), and included statements: (a) *Overall, it was easy to use the new GONAL-f Pen;* (b) *I am confident that I injected the right dose with the new GONAL-f Pen*; (c) *If this is a second cycle, compared to the injectable device from previous cycle, the new GONAL-f pen is more comfortable to use*; (d) *Based on my experience with the new GONAL-f pen, I would undergo another cycle using the same device*; (e) *The use of the new GONAL-f pen did not significantly interfere with my daily or work-related activities* (Supplementary Table 1). Patients had to select one of five answers ranging from “*strongly agree*” to “*strongly disagree*”. Since the final number of patients classified as at risk of OHSS was not available for the historical cohort, we used biomarkers of OHSS (AMH, AFC, basal FSH, basal LH, E2) and patient characteristics (BMI, age) to identify patients at risk of OHSS in both groups and then matched patients at risk of OHSS from the study group with patients at risk of OHSS from the control group. Patients at risk for OHSS were identified by independent investigator assessment, based on a biomarker prediction model.

All adverse events were summarized using number and proportion. The incidence of adverse events was reported as the number of patients reporting an adverse event (multiple occurrences of the same adverse event in an individual were counted only once in the number and proportion]). The incidence rate and severity of OHSS were assessed and reported for both the study and control groups. The severity of OHSS was classified based on the UK Royal College of Obstetricians and Gynaecologists 2016 guidelines [23].

### Statistical analysis

Overall, 200 patients considered at risk of OHSS were needed to detect a 10% difference between the two treatment groups (with 80% power) in the reduction of mean amount of r-hFSH (IU) administered per oocyte retrieved; therefore, a total enrolment of 2000 patients was planned to recruit 200 patients at risk of OHSS (assuming that 10% of ART patients would be considered to be at risk of OHSS).

Matched pairs for the primary endpoint analysis between the study and control groups were determined using a Greedy Algorithm [24]. This method takes into account confounders to create matched pairs with similar confounding factors for subjects belonging to the same pair. Matching (1:1) was performed based on potential risk factors for OHSS, including biomarkers (AMH >3.6 ng/mL, AFC >20, baseline FSH, baseline LH, E2) and patient characteristics (BMI, age). Missing observations in any of the risk factors were not considered while matching; therefore, only 393 patients in the control group who had reported AMH levels at baseline were eligible for matching with patients in the study group. The matched pairs were determined on the weighted sum of the absolute differences between the cases and the controls. Descriptive statistics were used to summarize patient characteristics, secondary endpoints, and safety in the overall population. In addition to descriptive statistics for the matched pairs, p-values from t-tests were calculated to assess the balance after matching for each of the biomarkers and patient characteristics used in matching.

A multiple logistic regression analysis was performed to estimate the impact of biomarkers and subject characteristics on the risk of OHSS for combined cohorts. The dependent variable was OHSS. The independent variables were study (historical/prospective), age (years), BMI (kg/m^2^), AMH level (ng/ml), AFC, FSH level at baseline (IU/L), LH level (IU/L) at baseline, and E2 level (pg/mL) on the day of hCG administration. The results of multiple logistic regression analysis were reported with regression coefficient, standard error, p-value, and odds ratio with 95% CI, for the subject characteristics and biomarkers that were statistically significant.

## Results

### Patient distribution, demographics and baseline characteristics

**Fig. 1:**
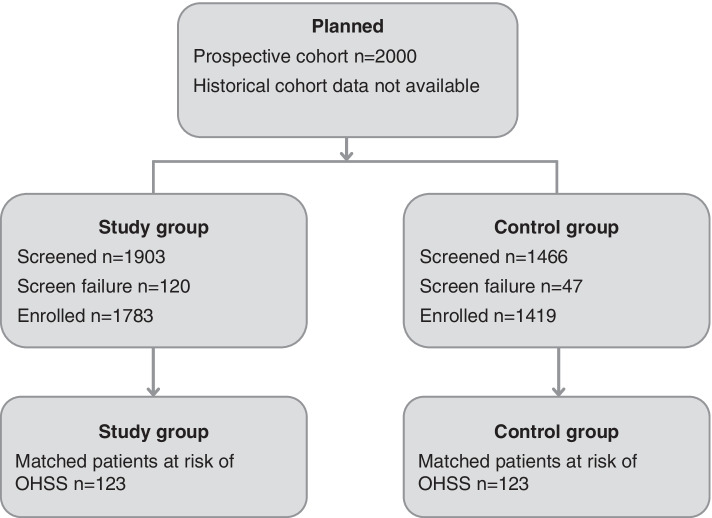
Patient distribution

Overall, 1783 patients were enrolled in the study group and 1419 patients were eligible for the analysis from the control group (Fig. 1). Demographic characteristics for both the study and control groups are presented in Table 2. In the study group 151/1783 patients (8.5%) had undergone a previous ART cycle (these data were not available for the control group).

**Table 2:** Patient demographics and baseline characteristics

	Full patient groups		At-risk for OHSS (matched groups)		
	Study group (N=1783)	Control group (N=1419)	Study group (n=123)	Control group (n=123)	p-value*
Age, years (mean ± SD)	31.2 ± 4.0	31.4 ± 4.1	29.3 ± 4.0	32.4 ± 4.1	<0.0001
Age categories					
≤35 years, n (%)	1522 (85.4)	1161 (81.8)	117 (95.1)	90 (73.2)	
>35 years, n (%)	261 (14.6)	258 (18.2)	6 (4.9)	33 (26.8)	
Weight (kg)	53.9 ± 7.7	55.7 ± 8.2	53.4 ± 7.8	55.6 ± 8.3	0.0168
Height (cm)	158.9 ± 5.4	159.4 ± 5.8	158.1 ± 5.0	160.2 ± 4.4	0.0003
BMI (kg/m^2^)	21.3 ± 2.7	21.9 ± 3.1	21.3 ± 2.7	21.7 ± 3.1	0.1876
ART treatment history					
First ART cycle, n (%)	1632 (91.5)	N/A	116 (94.3)	N/A	
Second ART cycle, n (%)	151 (8.5)	N/A	7 (5.7)	N/A	
FSH level at baseline					
n (%)	1355 (76.0)	1348 (95.0)	119 (96.7)	123 (100.0)	
mean ± SD (IU/L)	5.9 ± 2.5	6.0 ± 2.1	4.5 ± 2.3	5.4 ± 2.4	0.0012
LH level at baseline					
n (%)	1407 (78.9)	1268 (89.4)	119 (96.7)	123 (100.0)	
mean ± SD (IU/L)	4.0 ± 2.8	3.9 ± 2.7	3.2 ± 2.3	3.3 ± 2.3	0.2709
AMH level at baseline					
n (%)	1382 (77.5)	393 (27.7)	123 (100.0)	123 (100.0)	
mean ± SD (ng/mL)	5.6 ± 4.4	3.8 ± 2.5	7.6 ± 3.4	3.1 ± 2.8	<0.0001
AFC at baseline					
n (%)	1525 (85.5)	1128 (79.5)	123 (100.0)	123 (100.0)	
mean ± SD	12.4 ± 7.3	10.9 ± 5.1	14.3 ± 7.8	7.9 ± 4.5	<0.0001
Down-regulation regimen, n (%)					
Available	1783 (100.0)	1419 (100.0)	105 (85.4)	123 (100)	
Missing	0 (0.0)	0 (0.0)	18 (14.6)	0 (0.0)	
GnRH agonist	1115 (62.5)	1419 (100)	98 (93.3)	123 (100.0)	
GnRH antagonist	659 (37.0)	0 (0.0)	7 ( 6.7)		
Both	5 (0.3)	0 (0.0)			
Other	4 (0.2)	0 (0.0)			
Ovulation trigger					
n (%)	1747 (98.0)	1299 (91.5)	123 (100.0)	123 (100.0)	
Missing, n (%)	36 (2.0)	120 (8.5)			
hCG	··	1299 (100.0)		123 (100.0)	
rhCG	1556 (89.1)	··	115 (93.5)		
uhCG	144 (8.2)	··			
Agonist	35 (2.0)	··	7 (5.7)		
Combination^a^	12 (0.7)	··	1 (0.8)		
r-hLH co-administration, n (%)	333 (18.7)	366 (25.8)	40 (32.5)	26 (21.1)	

*AFC *antral follicle count; *AMH *anti-Müllerian hormone; *ART *assisted reproductive technology; *BMI *body mass index; *FSH *follicle-stimulating hormone; *GnRH* gonadotropin-releasing hormone; *LH* luteinizing hormone; *OHSS* ovarian hyperstimulation syndrome; *PCOS* polycystic ovary syndrome; *r-hLH* recombinant human luteinizing hormone; *SD* standard deviation; *uhCG* urinary human chorionic gonadotropin

*P-values from t-tests for the biomarkers and patient characteristics used in matching

^a^Combinations: (rhCG, agonist), (rhCG, agonist, others), (uhCG, agonist)

Data are presented as mean ± SD unless stated otherwise

In the study group, 62.5% (1115/1783) of patients were on a GnRH agonist protocol and 37.0% (659/1783) on an antagonist protocol; in the control group, all patients received a GnRH agonist (Table 2). In the study group, 18.7% (333/1783) of patients were on LH supplementation and 25.8% (366/1419) were on LH supplementation in the control group (Table 2). Data on ovulation trigger were available for 1747 (98.0%) of patients in the study group, of whom ovulation was triggered for 1556 patients with recombinant hCG, 144 with urinary hCG, 35 with an agonist, and 12 with a combination of triggers. All patients in the control group (data available for 1299 patients [92.0%]) received a hCG trigger (Table 2). Out of 333 patients on LH supplementation in the study group, 88 patients (26.4%) received r-hFSH from the pen injector in a fixed 2:1 combination with r-hLH (Pergoveris®; follitropin alfa/lutropin alfa; Merck Healthcare KGaA, Darmstadt, Germany), of whom three patients were included in the matched pair analysis. The other patients that received LH supplementation had either a separate injection with r-hLH (Luveris®; lutropin alfa; Merck Healthcare KGaA, Darmstadt, Germany) or a highly purified human menopausal gonadotropin containing LH-activity (HP-hMG).

A total of 397/1783 (22.3%) patients were identified to be at risk of OHSS in the study group based on the investigator’s judgement. The data regarding individual risk factors contributing to the decision on whether a patient was at risk of OHSS were available for 365 cases: AMH >3.6 ng/mL (n = 215), AFC >20 (n = 41), FSH (n = 49), polycystic ovary syndrome (PCOS) (n = 4), previous hyperstimulation (n = 1), other (n = 55). On the basis of baseline biomarkers and patient characteristics, 123 patients at risk of OHSS in the study group were matched with the 123 patients at risk of OHSS in the control group. P-values from t-tests are provided to compare the biomarkers and patient characteristics at baseline used for matching (Table 2). The r-hFSH starting dose for approximately 10.0% (24/123) of patients in the study group was determined using the small dose pen (12.5 IU increments); however, this small dose pen was not used by the control group (Table 3).

**Table 3:** r-hFSH starting dose in those at risk of OHSS (matched pairs)

r-hFSH starting dose (IU)	Study group (N = 123)	Control group (N = 123)
112.5	10 (8.1%)	0 (0.0%)
125.0	2 (1.6%)	0 (0.0%)
150.0	65 (52.8%)	33 (26.8%)
185.0	0 (0.0%)	1 (0.8%)
187.5	5 (4.1%)	23 (18.7%)
200.0	9 (7.3%)	0 (0.0%)
225.0	25 (20.3%)	54 (43.9%)
250.0	3 (2.4%)	0 (0.0%)
262.5	0 (0.0%)	1 (0.8%)
300.0	4 (3.3%)	11 (8.9%)

Data presented as n (%)

### Primary endpoint

**Fig. 2:**
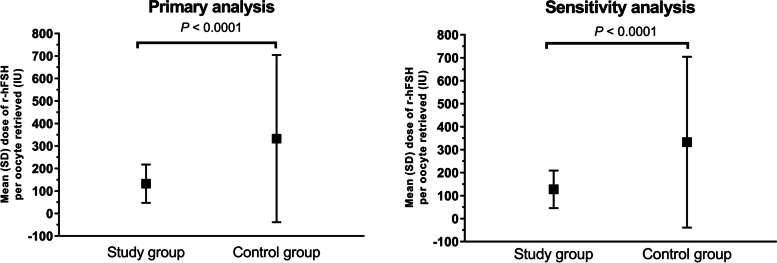
Mean amount of r-hFSH (IU) administered per oocyte retrieved for matched pairs of patients (n = 123) at risk of OHSS – primary analysis (all patients, irrespective of GnRH protocol) and sensitivity analysis (in patients receiving GnRH agonist)

The main analysis of the primary endpoint showed that the mean (SD) total dose of r-hFSH per oocyte retrieved was significantly lower in the study group (132.5 [85.2] IU) compared with the control group (332.7 [371.6] IU, *P* < 0.0001) (Fig. 2). The median (Q1–Q3) total dose of r-hFSH per oocyte retrieved was 107.1 (75.0–166.7) IU in the study group and 202.5 (125.0–375.0) IU in the control group. The same outcome was observed in the sensitivity analysis: the mean (SD) total dose of r-hFSH per oocyte retrieved was significantly lower in the study group (127. 5 [81.9] IU) compared with the control group (332.7 [371. 6] IU, *P* < 0.0001); the median (Q1–Q3) total dose of r-hFSH per oocyte retrieved was 102.3 (69.6–160.7) IU in the study group and 202.5 (125.0–375.0) IU in the control group.

### Secondary endpoints

Mean initial, total, and daily doses of r-hFSH evaluated for overall cohorts were numerically lower in the study group than in the control group (Table 4). Mean (SD) duration of r-hFSH treatment was 9.6 (1.7) days in the study group and 10.2 (1.6) days in the control group (Table 4). Overall, in the study group, 47.2% (841/1783) of patients required a dose adjustment of any amount (76 [61.8%] in the matched pairs), with 5.9% (105/1783) of patients requiring at least one dose adjustment of <37.5 IU (6 [4.9%] patients in the matched pairs) (Table 4). Of the patients who required a dose adjustment of <37.5 IU, most required a dose decrease. Data on dose adjustments were not available for the control group. Mean E2 level on the day of hCG administration was higher in the study group compared with the control group. Mean E2 level on the day of hCG administration was also higher with a GnRH agonist (4186.6 [2345.9] pg/mL) compared with a GnRH antagonist (3723.3 [3314.5] pg/mL) in the study group (Table 4).

**Table 4:** Secondary endpoints

	Full patient groups		At-risk for OHSS (matched groups)	
	Study group	Control group	Study group	Control group
	(N=1783)	(N=1419)	(N=123)	(N=123)
E2 level on the day of hCG administration^a^				
n (%)	1504 (84.4)	1011 (71.2)	53 (43.1)	113 (91.9)
Missing, n (%)	279 (15.6)	408 (28.8)	70 (56.9)	10 (8.1)
mean ± SD (pg/mL)	4058.3 ± 2663.5	3291.8 ± 2313.9	3317.3 ± 1116.6	2354.9 ± 1236.9
GONAL-f administration				
Initial dose of r-hFSH				
n (%)	1783 (100.0)	1419 (100.0)	123 (100.0)	123 (100.0)
Missing, n (%)	0	0	0	0
mean ± SD (IU)	191.7 ± 59.7	217.3 ± 60.2	174.4 ± 43.7	204.6 ±43.6
Cycle day on which r-hFSH treatment was initiated				
n (%)	1776 (99.6)	1416 (99.8)	123 (100.0)	123 (100.0)
Missing, n (%)	7 (0.4)	3 (0.2)	0	0
mean ± SD (days)	5.4 ± 3.4	4.1 ± 2.7	6.9 ± 3.1	3.4 ± 2.4
Total r-hFSH dose				
n (%)	1783 (100.0)	1419 (100.0)	123 (100.0)	123 (100.0)
Missing, n (%)	0	0	0	0
mean ± SD (IU)	1848.4 ± 700.5	2237.8 ± 772.6	1763.1 ± 587.2	2234.0 ± 782.2
Daily r-hFSH dose				
n (%)	1783 (100.0)	1419 (100.0)	123 (100.0)	123 (100.0)
Missing, n (%)	0	0	0	0
mean ± SD (IU)	314.6 ± 182.1	326.8 ± 201.3	172.4 ± 45.6	213.5 ± 50.0
r-hFSH dose per oocyte				
n (%)	1758 (98.6)	1408 (99.2)	123 (100.0)	123 (100.0)
Missing or zero oocytes retrieved, n (%)	25	11	0	0
mean ± SD (IU)	206.1 ± 253.2	268.8 ± 313.6	132.5 ± 85.2	332.7 ± 371. 6
Duration of r-hFSH treatment				
n (%)	1783 (100.0)	1419 (100.0)	123 (100.0)	123 (100.0)
Missing, n (%)	0	0	0	0
mean ± SD (days)	9.6 ± 1.7	10.2 ± 1. 6	10.1 ± 1.4	10.3 ± 1. 5
Patients with ≥1 dose adjustment (any amount)	841 (47.2)	Data not available	76 (61.8)	Data not available
Patients with ≥1 dose adjustment of <37.5 IU, n (%)	105 (5.9)		6 (4.9)	
Outcomes				
Implantation rate, %	30.0	20.6	38.4	12.7
Clinical pregnancy rate, %				
per embryo transfer cycle	50.3	40.7	56.4 (31/55)	26.1 (30/115)
per initiated cycle	35.3	37.8	25.2 (31/123)	24.4 (30/123)
Live birth rate, %				
per embryo transfer cycle	43.8	34	49.1 (27/55)	20.9 (24/115)
per initiated cycle	30.7	31.6	22.0 (27/123)	19.5 (24/123)
Multiple pregnancy rate, %				
per embryo transfer cycle	16.2	12.6	21.8 (12/55)	4.3 (5/115)
per initiated cycle	11.4	11.7	9.8 (12/123)	4.1 (5/123)
Cycle cancellation rate, %	27.3	7.1	20.3 (25/123)	6.5 (8/123)
Excessive response (risk of OHSS)^b^		55 (54.5)	0.0 (0.0)	3.0 (37.5)
Inadequate response^bc^	170 (34.9)	28 (27.7)	13.0 (52.0)	3.0 (37.5)
Other^d^	210 (43.1)	18 (17.8)	12.0 (48.0)	2.0 (25.0)

*E2 *estradiol; *GnRH *gonadotropin-releasing hormone; *hCG *human chorionic gonadotropin; *LH *luteinizing hormone; *N/A *not applicable; *OHSS *ovarian hyperstimulation syndrome; *r-hFSH *recombinant human follicle-stimulating hormone; *SD *standard deviation

^a^E2 values inconsistent with embryo transfer, i.e. ≥5000 pg/mL, for both the study group (n = 53) and control group (n = 113) were set to missing

^b^Denominator for % calculation was total number of patients who had a cancelled cycle: n = 487 in the study group, n = 101 in the control group

^c^Reasons for an inadequate response in study group were lack of ovarian response to stimulation treatment (n = 115), no fertilization (n = 53), no oocytes retrieved (n = 2)

^d^In the study group, the main reason listed as ‘other’ (n = 210) was ‘frozen embryo transfer’ (n = 161); reasons for frozen embryo transfers were not detailed in the case report form. The other 49 cases were related to reduced implantation potential, insufficient or low quality eggs/embryos, or patient preference

Other secondary outcomes, including implantation rates, clinical pregnancy rates per embryo transfer cycle, live birth rates per cycle, and multiple pregnancy rates per cycle, were numerically higher in the study group compared with the control group (Table 4). Cycle cancellation rate for the study group was 27.3% (487/1783), with the main reasons for cancellation being “other” (n = 210), “inadequate response” (n = 170), and “excessive response” (n = 107) (Table 4). In the study group, owing to data entry errors in several centers, 161 cycles were misclassified in the category “other” as cancelled cycles, but were actually “freeze-all” cycles, with the frozen embryo transfer taking place in subsequent cycle(s). After exclusion of these 161 misclassified cycles, the corrected cycle cancellation rate was 18.0% (326/1783) in the study group. In the control group, the overall cycle cancellation rate was 7.1% (101/1419) (n = 55 due to “excessive response”, n = 28 due to “inadequate response” and n = 18 due to “other” reasons) (Table 4). Data for frozen embryo cycles were not available for the control group.

Multiple logistic regression analysis of the biomarkers to predict risk of OHSS for the combined population of patients from the study and control groups showed that patients with an AFC of ≤20 were at lower risk of developing OHSS when compared with patients with an AFC of >20 (*P* = 0.033). Patients with a BMI of 18.5–30.0 kg/m^2^ were at lower risk of developing OHSS compared with patients with a BMI of <18.5 kg/m^2^ (*P* = 0.018) (Table 5).

**Table 5:** Significant risk factors of OHSS in combined cohorts

Independent variable	Category	Regression Coefficient	Standard Error	P-value	Odds Ratio (95% CI)
N	3202				
AFC	>20 ≤20	-1.1	0.5	0.0333	1.00 0.32 (0.1–0.91)
BMI (kg/m^2^)	<18.5 18.5–30.0	-1.2	0.5	0.0178	1.00 0.30 (0.11–0.81)

*AFC* antral follicle count; *BMI* body mass index; *OHSS* ovarian hyperstimulation syndrome; *CI* confidence interval

The patient satisfaction and quality of life evaluation data were available for 522 (29.3%) patients in the study group. Most patients “*strongly agreed”* or “*agreed”* that, overall, it was easy to use the new pen (511/522, 98.0%), they were confident that they injected the right dose (509/522, 98.0%), they would undergo another cycle using the same device (476/522, 91.0%), and the use of the GONAL-f pen injector did not significantly interfere with their daily or work-related activities (494/522, 95.0%). Out of 522 subjects, 61 undergoing a second cycle “*strongly agreed*” or “*agreed*” that the redesigned GONAL-f pen injector was more comfortable to use than the other injection devices used in a previous cycle.

### OHSS and AEs

OHSS was reported in 1.5% (27/1783) of patients in the study group and in 4.0% (57/1419) of patients in the control group (*P* < 0.0001; Table 6). Most of the OHSS events in the study group were of mild-to-moderate severity. Patients with clinical pregnancy in the study group and control group (50.3% and 40.7%, respectively, per embryo transfer and 35.3% and 37.8%, respectively, per initiated cycle) may be considered at risk for late onset OHSS (Table 4). However, these numbers should be interpreted with caution as there was a higher rate of cycle cancellation in the study group (27.3%) than in the control group (7.1%) (Table 4).

**Table 6:** OHSS events and severity

	Study group (N = 1783)	Control group (N = 1419)
OHSS events, n (%) P-value^a^	27 (1.5)	57 (4.0) *P* < 0.0001
OHSS severity, n (%) Mild/Grade I Moderate/Grade II Severe/Grade III	12 (0.7) 13 (0.7) 2 (0.1)	32 (2.3) 20 (1.4) 5 (0.4)

*OHSS* ovarian hyperstimulation syndrome

^a^Chi square test was used to calculate the p-value

Of 1783 subjects in the study group, a total of 89 subjects (5.0%) reported at least one AE. The most common AEs (>1% in the overall group) were OHSS (n = 27 [1.5%]) and spontaneous abortion (n = 35 [2.0%]) (Supplementary Table 2). Overall, 33 (1.9%) of patients reported at least one serious AE (SAE), with most requiring hospitalization (Supplementary Table 2). The most common SAEs were ectopic pregnancy (n = 10; 0.6%), OHSS (n = 7; 0.4%), and spontaneous abortion (n = 6; 0.3%) (Supplementary Table 2). The only treatment-related AE observed was OHSS (n = 26; 1.5%). AEs leading to study termination were reported in 1.1% of patients (n = 20) in the study group (Supplementary Table 2). No deaths were reported in the study group or control group.

## Discussion

In this study, the mean total dose of r-hFSH administered per oocyte retrieved was significantly lower in patients at risk of OHSS who used the r-hFSH pen injector with a 12.5 IU dose increment dial compared with matched patients who used other r-hFSH injection devices only allowing larger (≥37.5 IU) dose increments. The small dose pen (12.5 IU increments) was used to determine starting dose for 10% of the study group, and approximately 5% of total study patients. While these figures appear low, it is important to note that the small dose pen is a relatively recent development, and real-world data will continue to be collected as providers gain more experience. The ability to adjust r-hFSH dose in smaller increments (12.5 IU) allowed for treatment to be more tailored to the individual and resulted in the lower mean total dose of r-hFSH administered per oocyte retrieved observed.

In an analysis of the Global Safety Database of Merck Healthcare KGaA (including more than 20 years of data) and a systematic review of the reported incidence of OHSS in a population treated with GONAL-f, the reported rate for OHSS was 6.7 per 100,000 (0.007%) for all treatments started, and the review of the literature showed that the reported rate was 5.9% [25]. Therefore, the OHSS incidence rates reported in our study population (study group 1.5% and control group 4.0%) with broad inclusion criteria in both cohorts, fall within the incidence rates reported in clinical studies and in registries. The rate of severe OHSS as a function of all OHSS cases reported by Velthuis et al. was 3.7% (10 cases out of 272 in total) [25]. However, only 33 of these studies ranked OHSS according to severity, and the parameters used to classify the severity of OHSS were only reported in 15 of these 33 articles. This exemplifies the difficulties in reporting on the severity of OHSS, due to the different classification systems available and how these have developed over time. By contrast, the severity of OHSS in the results reported here were classified according to the UK Royal College of Obstetricians and Gynaecologists 2016 guidelines [23], which provides increased confidence in the consistency of the classification of OHSS severity.

During ovarian stimulation, the risk of OHSS increases with the number of follicles recruited. Therefore, the aim is to identify the optimal r-hFSH starting dose to recruit follicles, but with minimal risk of hyper-response and associated severe OHSS. The function of incremental dosing with the r-hFSH pen injector has been developed in response to the need for more streamlined treatment approaches for patients at risk of OHSS [26, 27]. The PIVET and CONSORT algorithms aim to individualize gonadotropin dosing, with starting doses based on patient characteristics and predicted ovarian response [9–11]. The application of the PIVET and CONSORT dosing algorithms has been enabled by r-hFSH pens that allow for smaller incremental dose adjustment. Compared with conventional dosing, introduction of the PIVET and CONSORT algorithms optimized the number of oocytes retrieved while significantly reducing daily and total dose of r-hFSH, the incidence of referral for increased monitoring and treatment for OHSS, and the incidence of freeze-all cycles, without diminishing the clinical pregnancy rate [9–11]. The PIVET algorithm re-designed specifically for the GONAL-f pen with a 12.5 IU dose increment dial was validated on the basis of effectiveness and safety in terms of OHSS [11]. Compared with the use of the PIVET algorithm for the Puregon® pen (Merck Sharp & Dohme, Sydney, Australia) with a 8.3 IU dose increment dial, the use of the GONAL-f pen demonstrated similar pregnancy and live birth rates [11]. The results obtained using the CONSORT and PIVET algorithms enable comparison of OHSS rates reported using r-hFSH-alfa injection devices with small dosing increments in regions other than the ones reported in this manuscript. In the CONSORT study, which included a cohort of predicted normo-ovulatory women in nine European countries and one centre in Chile, the OHSS rate reported for women with an individualized starting dose was 6.3% (6/96) compared with 12.5% (13/104) in women with a standardized starting dose [9]. Additionally, an analysis of outcomes for women treated at fertility centres in Australia who were assigned starting doses according to the PIVET algorithm reported an OHSS rate of 0.3% (nine cases out of 2,822 stimulation cycles), all of which were attributed to non-compliance with PIVET algorithm protocols and regimens [11]. With increasing experience of devices that allow adjustment of the dose of r-hFSH-alfa in small increments, we anticipate that a clearer picture will emerge on the incidence of OHSS.In our study, the mean starting dose, total dose, and daily doses of r-hFSH were numerically lower in the study group compared with the control group. This observation is in line with results of a previous study in patients (N = 200) with normal ovarian response using the GONAL-f pen injector [9], in which two patient groups receiving either a standard dose of r-hFSH (150 IU per day) or individualized doses based on the CONSORT algorithm (112.5, 150.0, 187.5, 225.0, 300.0, or 450.0 IU per day) were compared. Significantly lower mean daily (121.5 vs. 167.4 IU; *P* < 0.001) and total (1288.5 vs. 1810.0 IU; *P* < 0.001) doses of r-hFSH were reported when starting gonadotropin dose was individualized based on the CONSORT algorithm versus standard dosing [9]. Clinical pregnancy was comparable in both the CONSORT group and standard dosing group (36.0% vs. 35.5%), but a significant reduction in OHSS was reported in the CONSORT group compared with the standard dose group. The value of individualized dosing and small dose adjustments to mitigate the risk of OHSS has also been confirmed in patients with a predicted hyper-response to ovarian stimulation. A study by Oudshoorn et al. compared clinical outcomes in 521 patients undergoing ovarian stimulation with a reduced r-hFSH dose (100.0 IU/day) and a standard dose (150.0 IU/day), while allowing dose adjustments of 25 IU throughout the treatment cycle. There was a significant reduction in the occurrence of any grade of OHSS with a lower dose compared to standard dose (5.2% vs. 11.8%, *P* = 0.001) [8].

Individualization of ovarian stimulation includes both selection of the starting gonadotropin dose, as well as intra-cycle dose adjustments, both of which are important to achieve an optimal outcome of fertility treatment [3, 14]. While dose adjustment is common in routine clinical practice, reported in up to 45.0% of cycles [3, 16, 28], selection of the optimal starting dose may potentially reduce the need for further dose adjustments during the cycle. This was reported in a study assessing application of the PIVET algorithm, in which precise selection of the starting dose using the algorithm meant that 79.1% of patients didn’t require any further adjustments during the treatment [11]. This was in accordance with the results observed in our study, where only about 6.0% of patients required at least one dose adjustment of <37.5 IU during the treatment cycle, mostly a dose decrease (Table 5). Nevertheless, the benefits of combining optimal starting dose selection strategies and intra-cycle dose adjustments in respect to OHSS risk reduction are evident, and individualized treatment decisions should be based on both pre-treatment evaluation and monitoring during the treatment.

The regression analysis of the biomarkers to predict risk of OHSS in our study showed that AFC >20 and low BMI were associated with an increased risk of OHSS. In a systematic review, La Marca and Sunkara (2014) reported an even lower AFC cut-off of between 9 and 16 as a potential biomarker to predict hyper-response [29]. Similarly, BMI has been identified as one of the risk factors for OHSS by the American Society for Reproductive Medicine [12]. Specifically, two studies referenced in the ASRM guideline for the management of OHSS reported a correlation between a low BMI and the development of OHSS [30, 31]. BMI is also included as a prognostic indicator for ovarian response in the CONSORT algorithm, based on evidence from a meta-analysis including 1378 ART patients [32], and a PIVET algorithm [10]. In addition, a number of studies investigated a role of serum AMH levels in the prediction of ovarian response confirmed that high basal AMH (>3.3 ng/mL) on Day 3 is associated with an increased risk of developing OHSS [33–35].

Our study results showed that the r-hFSH pen evaluated in the study group is patient-friendly and easy to use. The majority of patients who completed the treatment satisfaction questionnaire agreed that, overall, the redesigned pen injector was easier and more comfortable to use than the devices they had used previously, which is supported by the findings from other studies evaluating the use of the r-hFSH pen injector [17, 19, 20]. The usability, engineering, and dose accuracy of the GONAL-f pen injector with a 12.5 IU dose dial feature was previously tested. It was demonstrated that it can be used safely and effectively and the dose can be injected with accuracy under a range of different conditions by patients with infertility and fertility nurses [17, 18, 36]. Further simulated-use and questionnaire-based studies confirmed that the pen injector was easy to use for patients and easy for fertility nurses to train with [19, 20, 37]. The optional patient questionnaire provided to patients in our study was similar to that used in the previous study [20]. Of the patients that filled in the questionnaire in our study, 98% were confident that they injected the right dose, which was comparable to the response provided in the previous study, where 94.0% agreed that they could administer the correct dose [20]. Finally, in a comparative evaluation of the use of four available r-hFSH pen injectors, the GONAL-f injector received the highest ratings and was preferred by both patients and nurses [22].

This study had some limitations. Both the prospective and historical studies were open-label and observational and therefore had inherent limitations in terms of susceptibility to bias. However, the main analysis and sensitivity analysis of the primary endpoint were performed using a matched pair analysis [24], a methodology designed to limit confounding factors between cohorts. Although the two cohorts were similar according to inclusion/exclusion criteria and baseline characteristics, some differences may still exist, as more countries were included in the historical study (control group) compared with the prospective study (study group). In addition, patient assessment and monitoring was performed according to the protocols at each of the participating centers, and the final decision on whether a patient should be classified as at risk of OHSS was made by the investigator. In the results for the matched-pair analysis, there is some imbalance in some of the values used to match patients in the matched-pair groups. It should be noted that the Greedy algorithm does not match pairs of patients based on individual parameters, but rather it matches pairs based on the weighted sum of the differences between the cases and the controls. Therefore, some imbalance may be expected in the results for specific parameters included in the matching process. Furthermore, as patients with missing values were not considered in the Greedy algorithm, the pool of patients to select in the control group was limited to only 393 patients (i.e., those who had a reported AMH level at baseline), and this may also have contributed to the imbalance in the results for this parameter in the matched-pair analysis. In the case of the AMH values reported here, this imbalance may actually strengthen our results, as the women in the study group had a higher risk of OHSS based on AMH levels than those in the control group, whereas a lower rate of OHSS was reported in this group.

The use of the long GnRH agonist protocol in the historical control group reflects the clinical practice in this region at that time, with the majority of patients/cycles being treated with agonists instead of antagonists. More recent insights and guidelines [7, 12] have led to the conclusion that OHSS risk can be reduced with antagonist use, which is why the study group only received antagonists. It is clear that the use of only antagonists in the study group, together with the 12.5 IU increment pen, have both contributed to a lower OHSS risk, but this lower OHSS risk needs to be considered with care, as it is a secondary exploratory outcome, and many factors contribute to the ultimate OHSS risk, including the cancellation policy. Furthermore, in the additional analysis of the 123 matched pairs, 93.3% of the participants in the study group for whom these data were available received an agonist protocol (compared with 100% of the control group), showing that these two cohorts were well matched with regard to the GnRH down-regulation protocol used.

In total, in the study group, a higher proportion of patients (487/1783 [27.0%], or 326/1783 [18.0%] patients after exclusion of 161 misclassified cycles) had cycle cancellations when compared with the control group (7.1%). Most of the cancelled cycles were due to “other” reasons in the study group (mostly “freeze-all” cycles, with the frozen embryo transfer taking place in a subsequent cycle), whereas most of the cancelled cycles were due to excessive response in the historical cohort. Embryo freezing was dependent on the clinical practice used in the centers at the time of the study, and the decision was made by the investigators and patients. The exact reasons for frozen embryo transfers could not be retrieved from all of the participating centers; therefore, these data were not reported.

Other limitations included attrition bias due to subjects lost to follow-up and overall patient recall regarding the satisfaction in relation to usability and quality of life. A further limitation of the study is that data analyses presented here were not adjusted based on the 88 patients who received r-hFSH from the pen injector in the 2:1 fixed combination with r-hLH (Pergoveris), of whom three patients were included in the matched pair analysis.

## Conclusions

This study assessed dosing characteristics and ovarian response in an Asian population and demonstrated that, compared with the older r-hFSH injecting devices, use of the r-hFSH pen injector with 12.5 IU increments was associated with the reduced total dose of r-hFSH used per oocyte retrieved in patients at risk of OHSS. Individualized dosing was associated with comparable clinical outcomes and a lower incidence of OHSS. Furthermore, the regression analysis of the biomarkers used to identify risk of OHSS showed that AFC >20 and BMI <20 kg/m^2^ correlate with the risk of developing OHSS. The results from this study support the need for more real-world studies to evaluate ovarian stimulation protocols and dosing characteristics with the aim to improve clinical outcomes and minimize OHSS and cycle cancellation in Asian patients.

## Supplementary Information

**Additional file 1.**

## Data Availability

Any requests for data by qualified scientific and medical researchers for legitimate research purposes will be subject to Merck KGaA’s Data Sharing Policy. All requests should be submitted in writing to Merck KGaA’s data sharing portal https://www.merckgroup.com/en/research/our-approach-to-research-and-development/healthcare/clinical-trials/commitment-responsible-data-sharing.html. When Merck KGaA has a co-research, co-development, or co-marketing or co-promotion agreement, or when the product has been out-licensed, the responsibility for disclosure might be dependent on the agreement between parties. Under these circumstances, Merck KGaA will endeavour to gain agreement to share data in response to requests.

## References

[1] Smitz J, Wolfenson C, Chappel S, Ruman J (2016). Follicle-Stimulating Hormone: A Review of Form and Function in the Treatment of Infertility. Reprod Sci.

[2] EMA. Summary of product characteristics: GONAL-f. 2009.

[3] Mol BW, Bossuyt PM, Sunkara SK, Garcia Velasco JA, Venetis C, Sakkas D (2018). Personalized ovarian stimulation for assisted reproductive technology: study design considerations to move from hype to added value for patients. Fertil Steril.

[4] Lunenfeld B, Bilger W, Longobardi S, Alam V, D’Hooghe T, Sunkara SK (2019). The Development of Gonadotropins for Clinical Use in the Treatment of Infertility. Front Endocrinol.

[5] Grisendi V, La Marca A (2017). Individualization of controlled ovarian stimulation in vitro fertilization using ovarian reserve markers. Minerva Ginecol.

[6] Popovic-Todorovic B, Racca A, Blockeel C (2018). Added value today of hormonal measurements in ovarian stimulation in gonadotropin-releasing hormone antagonist treatment cycle. Curr Opin Obstet Gynecol.

[7] ESHRE. Ovarian Stimulation for IVF/ICSI. 2019 [25 November, 2019]; Available from: https://www.eshre.eu/Guidelines-and-Legal/Guidelines/Ovarian-Stimulation-in-IVF-ICSI.

[8] Oudshoorn SC, van Tilborg TC, Eijkemans MJC, Oosterhuis GJE, Friederich J, van Hooff MHA, et al. Individualized versus standard FSH dosing in women starting IVF/ICSI: an RCT. Part 2: The predicted hyper responder. Hum Reprod. 2017;32(12):2506-14. 10.1093/humrep/dex319 29121269

[9] Olivennes F, Trew G, Borini A, Broekmans F, Arriagada P, Warne DW (2015). Randomized, controlled, open-label, non-inferiority study of the CONSORT algorithm for individualized dosing of follitropin alfa. Reprod Biomed Online.

[10] Yovich J, Stanger J, Hinchliffe P (2012). Targeted gonadotrophin stimulation using the PIVET algorithm markedly reduces the risk of OHSS. Reprod Biomed Online.

[11] Yovich JL, Alsbjerg B, Conceicao JL, Hinchliffe PM, Keane KN (2016). PIVET rFSH dosing algorithms for individualized controlled ovarian stimulation enables optimized pregnancy productivity rates and avoidance of ovarian hyperstimulation syndrome. Drug Des Devel Ther.

[12] ASRM (2016). Prevention and treatment of moderate and severe ovarian hyperstimulation syndrome: a guideline. Fertil Steril.

[13] Lensen SF, Wilkinson J, Leijdekkers JA, La Marca A, Mol BWJ, Marjoribanks J, et al. Individualised gonadotropin dose selection using markers of ovarian reserve for women undergoing in vitro fertilisation plus intracytoplasmic sperm injection (IVF/ICSI). Cochrane Database Systematic Reviews. 2018 ;2(2):Cd012693. 10.1002/14651858.CD012693.pub2 PMC6491064 29388198

[14] Lunenfeld B, Bilger W, Longobardi S, Kirsten J, D’Hooghe T, Sunkara SK (2019). Decision points for individualized hormonal stimulation with recombinant gonadotropins for treatment of women with infertility. Gynecol Endocrinol.

[15] La Marca A, Blockeel C, Bosch E, Fanchin R, Fatemi HM, Fauser BC, et al. Individualized FSH dosing improves safety and reduces iatrogenic poor response while maintaining live-birth rates. Hum Reprod. 2018;33(5):982-3. 10.1093/humrep/dey061 29596626

[16] Mahony M, Hayward B, Richter K, T DH, editors. Occurrence and characteristics of recombinant human follicle-stimulating hormone (r-hFSH) dose adjustments during ovarian stimulation in a real-world US database study of 33,962 IVF patient cycles. 34th Annual Meeting of the European Society of Human Reproduction and Embryology; 2018; Barcelona, Spain.

[17] Jeannerot F, Cusin A, Schertz J (2016). Dose accuracy of the redesigned follitropin alfa pen injector for infertility treatment. Expert Opin Drug Deliv.

[18] Jeannerot F, Stüdeli T, Gunther-LaVergne L, Hirning D, Schertz J (2016). Usability engineering study in the European Union of a redesigned follitropin alfa pen injector for infertility treatment. Expert Opin Drug Deliv.

[19] Schertz J, Worton H (2018). Nurse evaluation of the redesigned fertility pen injector: a questionnaire-based observational survey. Expert Opin Drug Deliv.

[20] Schertz J, Worton H (2017). Patient evaluation of the redesigned follitropin alfa pen injector. Expert Opin Drug Deliv.

[21] Abbotts C, Salgado-Braga C, Audibert-Gros C (2011). A redesigned follitropin alfa pen injector for infertility: results of a market research study. Patient Prefer Adherence.

[22] Longobardi S, Seidler A, Martins J, Beckers F, MacGillivray W, D’Hooghe T (2019). An evaluation of the use and handling errors of currently available recombinant human follicle-stimulating hormone pen injectors by women with infertility and fertility nurses. Expert Opin Drug Deliv.

[23] Mathur R, Drakeley A, Reine-Fenning N, Evbuomwan I, Hamoda H. Ovarian Hyperstimulation Syndrome, Management (Green-top Guideline No. 5). 2016. 10.1111/1471-0528.70195 41975565

[24] Bergstralh EJ, Kosanke JL. Computerized matching of cases to controls. Technical Report Series No. 56. Department of Health Science Research, Mayo Clinic, Rochester. 1995.

[25] Velthuis E, Hubbard J, Longobardi S, D’Hooghe T (2020). The Frequency of Ovarian Hyperstimulation Syndrome and Thromboembolism with Originator Recombinant Human Follitropin Alfa (GONAL-f) for Medically Assisted Reproduction: A Systematic Review. Adv Therapy.

[26] la Cour Freiesleben N, Gerds TA, Forman JL, Silver JD, Nyboe Andersen A, Popovic-Todorovic B (2011). Risk charts to identify low and excessive responders among first-cycle IVF/ICSI standard patients. Reprod Biomed Online.

[27] Popovic-Todorovic B, Loft A, Bredkjaeer HE, Bangsboll S, Nielsen IK, Andersen AN (2003). A prospective randomized clinical trial comparing an individual dose of recombinant FSH based on predictive factors versus a ‘standard’ dose of 150 IU/day in ‘standard’ patients undergoing IVF/ICSI treatment. Hum Reprod.

[28] Fatemi HM, Bilger W, Denis D, Griesinger G, La Marca A, Longobardi S, et al. Dose adjustment of follicle stimulating hormone (FSH) during ovarian stimulation as part of medically-assisted reproduction in clinical studies: a systematic review covering 10 years (2007–2017). Fertil Steril. 2020;114(3). 10.1186/s12958-021-00744-x PMC8112039 33975610

[29] La Marca A, Sunkara SK (2014). Individualization of controlled ovarian stimulation in IVF using ovarian reserve markers: from theory to practice. Hum Reprod Update.

[30] Danninger B, Brunner M, Obruca A, Feichtinger W (1996). Prediction of ovarian hyperstimulation syndrome by ultrasound volumetric assessment [corrected] of baseline ovarian volume prior to stimulation. Hum Reprod.

[31] Aramwit P, Pruksananonda K, Kasettratat N, Jammeechai K. Risk factors for ovarian hyperstimulation syndrome in Thai patients using gonadotropins for in vitro fertilization. Am J Health Syst Pharm. 2008;65(12):1148–53. 10.2146/ajhp070566 18541685

[32] Howles CM, Saunders H, Alam V, Engrand P (2006). Predictive factors and a corresponding treatment algorithm for controlled ovarian stimulation in patients treated with recombinant human follicle stimulating hormone (follitropin alfa) during assisted reproduction technology (ART) procedures. An analysis of 1378 patients. Curr Med Res Opin.

[33] Ocal P, Sahmay S, Cetin M, Irez T, Guralp O, Cepni I (2011). Serum anti-Mullerian hormone and antral follicle count as predictive markers of OHSS in ART cycles. J Assist Reprod Genet.

[34] Lee TH, Liu CH, Huang CC, Wu YL, Shih YT, Ho HN (2008). Serum anti-Müllerian hormone and estradiol levels as predictors of ovarian hyperstimulation syndrome in assisted reproduction technology cycles. Hum Reprod.

[35] Nardo LG, Gelbaya TA, Wilkinson H, Roberts SA, Yates A, Pemberton P (2009). Circulating basal anti-Mullerian hormone levels as predictor of ovarian response in women undergoing ovarian stimulation for in vitro fertilization. Fertil Steril.

[36] Mahony MC, Patterson P, Hayward B, North R, Green D (2015). Human factors engineering and design validation for the redesigned follitropin alfa pen injection device. Expert Opin Drug Deliv.

[37] Schertz J, Feilding B, Worton H. Patient and nurse evaluation of the improved follitropin alfa pen injector for infertility treatment. Fertility Sterility. 2016;106, No. 3, Supplement.

