# Supplementary material for: A comparative, observational study evaluating dosing characteristics and ovarian response using the recombinant human follicle-stimulating hormone pen injector with small-dose dial in assisted reproductive technologies treatment in Asia: IMPROVE study
Source: Reprod Biol Endocrinol. 2022 Jan 17;20:15. doi: 10.1186/s12958-021-00882-2 (PMC8762890; doi:10.1186/s12958-021-00882-2)
Supplement: Supplementary file 1 — Additional file 1. [file 12958_2021_882_MOESM1_ESM.docx]

Additional file 1: Supplementary (docx)

- Supplementary Table 1. Satisfaction on usability and quality of life patient questionnaire
- Supplementary Table 2. Adverse events (Study group)

# Appendix

***Supplementary Table 1****. Satisfaction on usability and quality of life patient questionnaire*

*
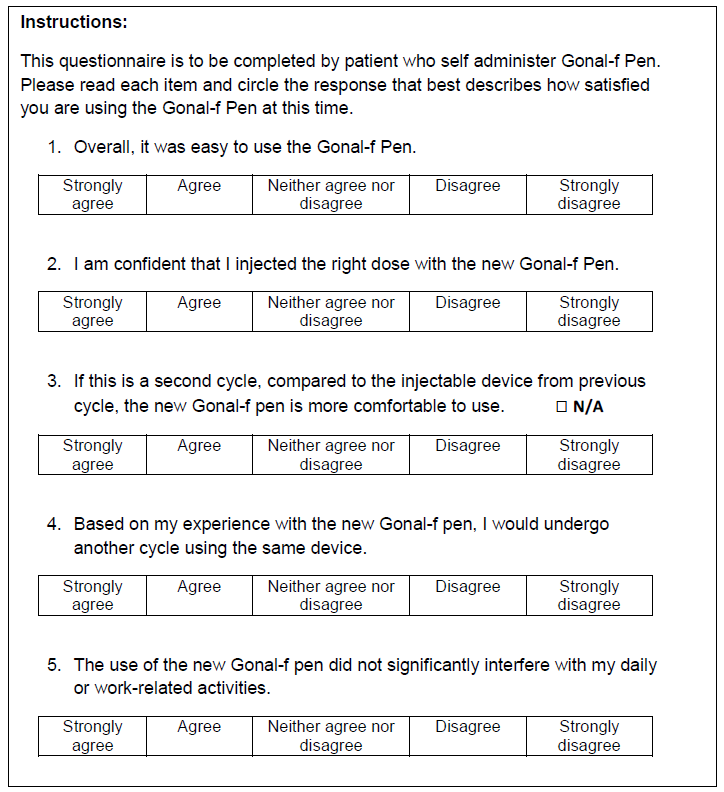
*

*Supplementary Table 2. Adverse events (Study group)*

|  | **Study group (N=1783)** |
| --- | --- |
| Number of patients with, n (%)  At least one AE  At least one SAE  At least one SAE requiring hospitalization  At least one treatment-related AE  At least one AE leading to study termination  At least one AE leading to death | 89 (5.0)  33 (1.9)  31 (1.7)  26 (1.5)  20 (1.1)  0 (0.0) |
| Congenital, familial and genetic disorders |  |
| Anencephaly | 1 (0.1) |
| Congenital heart disease | 1 (0.1) |
| Pulmonary hypoplasia | 1 (0.1) |
| General disorders and administration site conditions |  |
| Cyst | 1 (0.1) |
| Injury, poisoning and procedural complications |  |
| Post-procedural haemorrhage | 1 (0.1) |
| Pregnancy, puerperium and perinatal conditions |  |
| Abortion | 1 (0.1) |
| Spontaneous abortion | 35 (2.0) |
| Ectopic pregnancy | 10 (0.6) |
| Foetal death | 1 (0.1) |
| Hyperemesis gravidarum | 1 (0.1) |
| Induced labour | 2 (0.1) |
| Multiple pregnancy | 1 (0.1) |
| Premature delivery | 2 (0.1) |
| Premature labour | 4 (0.2) |
| Premature rupture of membranes | 1 (0.1) |
| Stillbirth | 3 (0.2) |
| Reproductive system and breast disorders |  |
| OHSS | 27 (1.5) |
| Skin and subcutaneous tissue disorders |  |
| Dermatitis | 1 (0.1) |
| Surgical and medical procedures |  |
| Selective abortion | 2 (0.1) |
| Most common SAEs  Ectopic pregnancy  OHSS  Spontaneous abortion | 10 (0.6)  7 (0.4)  6 (0.3) |

AE, adverse event; SAE, serious AE; OHSS, ovarian hyperstimulation syndrome.
